# *Primulina flexusa* sp. nov. (Gesneriaceae) from Guizhou Province, China

**DOI:** 10.3897/phytokeys.159.55386

**Published:** 2020-09-04

**Authors:** Tao Peng, Bo Pan, Stephen Maciejewski, Fang Wen

**Affiliations:** 1 Biodiversity Research Center, School of Life Sciences, Guizhou Normal University, CN-550025 Guiyang, China; 2 Guangxi Key Laboratory of Plant Conservation and Restoration Ecology in Karst Terrain, Guangxi Institute of Botany, Guangxi Zhuang Autonomous Region and Chinese Academy of Sciences, CN-541006 Guilin, China; 3 Gesneriad Conservation Center of China (GCCC), Guilin Botanical Garden, Guangxi Zhuang Autonomous Region and Chinese Academy of Sciences, CN-541006 Guilin, China; 4 The Gesneriad Society, 1122 East Pike Street, PMB 637 Seattle, WA 98122-3916 USA

**Keywords:** Cliff-dwelling, flora of Guizhou, limestone flora, lithophytic, taxonomy

## Abstract

The limestone regions of Yunnan-Guangxi-Guizhou in southern and southwestern China are regarded as some of biodiversity’s hotspots for China’s Gesneriaceae where numerous rare new species of *Primulina* have been, or are being, described over the past two decades. *Primulina flexusa*, a new lithophytic species of Gesneriaceae from a limestone hill in a Karst area, from Guizhou, China, is described here with color photographs. It is similar to *P. curvituba*, but can be easily distinguished by a combination of characteristics, especially in the shape and length of its capsule. We found only one population with approximately 100 mature individuals at the type locality. This new species is provisionally assessed as Critically Endangered [CR C1] by using IUCN criteria.

## Introduction

The vast majority of *Primulina* species have a straight funnelform-tubular to campanulate or cylindric corolla tube (Wang et al. 1990, 1998; Li and Wang 2005). They usually are not swollen or gibbous abaxially. However, there are some exceptions; for example, in the past decade, some newly published species of *Primulina* have an inflated corolla tube, like *P. crassituba* (W.T. Wang) Mich. Möller and A. Weber (Wang 1989), *P. carinata* Y.G. Wei, F. Wen & H.Z. Lü (Wen et al. 2014) and *P. inflata* Li.H. Yang & M.Z. Xu (Xu et al. 2020). Another rare corolla characteristic of *Primulina* is the curved tube. So far, before this new taxon was discovered, only one species of *Primulina* had a curved tube, namely *P. curvituba* B. Pan, L.H. Yang & M. Kang (Yang et al. 2017), out of a total of about 210 species of *Primulina* in China (Wen et al. 2019, 2020).

**Figure 1.**
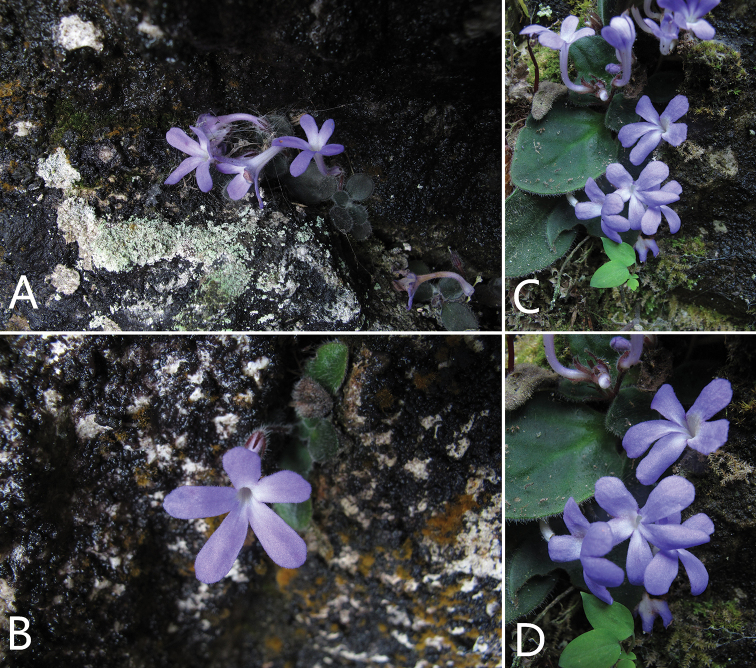
The flowering plants of *Primulina flexusa* growing in the crevice of limestone hill (type locality): **A–D** flowering plants from different aspects.

Some studies have suggested that moss may play a positive role in affecting the survival and growth of some *Primulina* species, for instance *P. tabacum* Hance (Ren et al. 2010a, b). Hence, one of the authors (PT), as a bryologist, is also very concerned about the biodiversity of *Primulina* when he carries out the fieldwork for bryophyte biodiversity and flora. Significantly, when PT investigated bryophyta in Duyun City, southern Guizhou Province in the autumn of 2017, an unknown species of Gesneriaceae, but one without flowers, was collected. The vegetative characters of the individuals of this species, e.g. their small but conspicuous rhizome, opposite leaves, fleshy, and elliptical but fragile leaf blade, indicated that it should be classified as a member of *Primulina*. It is somewhat similar to *P. tenuituba* (W.T. Wang) Yin Z. Wang, namely former *Deltocheilos tenuitubum* W.T. Wang (Wang 1981a) and *Chirita shennungjiaensis* W.T. Wang (Wang 1981b; Wang et al. 1990) because of the leaf blade morphology and shape. Several living individuals were then introduced into the nursery and gardens at the Gesneriad Conservation Center of China (GCCC), at the Guilin Botanical Garden, Chinese Academy of Sciences. After six months, those introduced and cultivated plants blossomed in April 2018. To our surprise, the gross morphology of flowers, such as their curved corolla tube, was obviously different from all other species of *Primulina*, except for *P. curvituba*. We revisited the locality immediately to collect flowering plants in the field (Figure 1). Surprisingly, when we showed photographs to the staff of the Guangxi Institute of Botany, one of the authors (BP) told us that he had been collecting the flowering specimens and living plants of this species back in 2016. Further literature study (e.g. Wang et al. 1998; Li and Wang 2005; Wei et al. 2010 and all recent published papers for new taxa of *Primulina*) and morphological comparison convinced us that it represents a new species to science, which is described and illustrated below.

## Taxonomic treatment

### Primulina flexusa

Taxon classificationPlantaeLamialesGesneriaceae

F. Wen, Tao Peng & B. Pan sp. nov.

8104A50C-2FEE-582A-867C-1DE9360BD24E

urn:lsid:ipni.org:names:77211381-1

Figures 1 , 2

#### Type.

China. Guizhou Province, Duyun City, Bamang town, Longtang village, 26.31N, 107.49E, altitude ca. 1040 m, 15 May 2016, *Bo Pan et al.*, *PB160425-01* (Holotype: IBK!; Isotype: IBK!).

#### Diagnosis.

The new species resembles *Primulina curvituba* in having a curved corolla but is easily distinguished from the latter by bracts oblong (*vs.* lanceolate), filament glabrous (*vs.* glandular-pubescent), ovary ovoid (*vs.* cylindrical), stigma slightly 2-parted at the apex (*vs.* undivided at apex) and capsule ovoid (*vs.* linear).

#### Description.

Herbs perennial, acaulescent. Leaves basal, 8–12, opposite, petiolate; petiole compressed, gradually broadened from the base to the upper, densely pilose, 5–13 × 1.5–3.5 mm; leaf blade ovate to broadly ovate, abaxial surface green to dark green, adaxial surface brownish-green to brownish-purple; 1.6–2.0 × 1.3–1.8 cm, pubescent and pilose on adaxial surface, puberulent on abaxial surface, base shallowly cordate to slightly cuneate, margin entire and ciliate, apex obtuse to nearly rounded; lateral veins ca. 3 on each side of the midrib, inconspicuous on adaxial surface, prominent on the abaxial surface. Cymes 2–4, 1–4-flowered; peduncle 1.5–2.4 cm long, ca. 1 mm in diameter, erect pubescent; bracts 2, opposite, oblong, ca. 3.8 × 0.7 mm, adaxially green and nearly glabrous, abaxially brownish-green to brownish-purple, densely pilose, the pilose hairs 0.8–1.2 mm long, margin entire, apex acute, bracteole ca. 2.4 × 0.5 mm, color and indumentum same as bracts. Pedicel 6.5–8 mm long, ca. 0.9 mm in diameter, puberulent. Calyx 5-parted from the base; segments equal, pale brown to brown, lanceolate, 4–5 × 0.7–0.9 mm, outside pale brown to greenish-brown, densely pubescent, inside greenish-brown, glabrous, margin entire, apex acute. Corolla 21–25 mm long, pale purple to purple, throat with two distinctly dark purple stripes respectively between each pair of abaxial lip lobes, outside covered with extremely short glandular-puberulent hairs, inside nearly glabrous; corolla tube infundibuliform, slightly curved downwards at base (ca. 4 mm from the base), then gradually bent forwards, 16–19 mm long, ca. 6 mm in diameter at the mouth, ca. 2.5 mm in diameter at the base; limb distinctly 2-lipped, adaxial lip 2-parted over 2/3 from the top of the adaxial lip, lobes nearly equal, broadly obovate, ca. 5 × 4 mm; abaxial lip 3-parted over 4/5 from the top of the abaxial lip, lobes slightly obliquely obovate, 7.5–8 × 3.9–5 mm. Stamens 2, adnate to ca 2.5 mm above the corolla base; anthers pale yellowish-brown, elliptic, ca. 1.5 × 1.0 mm, fused by entire adaxial surfaces; filaments linear, straight, ca. 3 mm long, white, glabrous; staminodes 3, adnate to ca. 3 mm above the corolla tube base, lateral ones ca. 3.5 mm long, the middle one ca. 0.7 mm long. Disc yellowish-green, annular, margin entire or sometimes slightly erose, ca. 0.4 mm high. Pistil ca. 5 mm long; ovary brownish-red, ovoid, ca. 1.4 mm long, ca. 0.9 mm in diameter, densely puberulent and glandular-puberulent; style white, 2.2–2.5 mm long, ca. 0.2 mm in diameter, sparsely glandular-puberulent. Stigma 1, translucent to white, its upper lobe lacking, lower lobe obtrapeziform, slightly 2-parted at apex, ca. 1 mm long, ca. 0.75 mm wide. Capsule ovoid, ca. 6.5 mm long, ca. 2.2 mm in diameter, densely puberulent.

#### Phenology.

Flowering occurs from April and fruiting from May to June.

#### Etymology.

The specific epithet ‘*flexusa*’ is derived from its curved corolla tube. The original epithet ‘*flexusa*’ derived from the Latin, ‘*flexus*’, means curved and slightly zigzagging.

#### Vernacular name.

Qū Guǎn Bào Chūn Jù Tái (Chinese pronunciation); 曲管报春苣苔 (Chinese name).

#### Distribution and habitat.

*Primulina flexusa* is hitherto only known from the type locality, Mangba town, Duyun City, Guizhou Province, Southwest China, growing on moist and shaded rocky crevice on the cliff in a subtropical evergreen seasonal rain forest, at an altitude of ca. 1040 m. All plants were growing in a damp and dark crevice of Karst cliff near a village.

#### Conservation status and IUCN RedList category.

Only a single population with ca. 100 mature individuals is known to exist at the type locality. All individuals were found growing in a large horizontal crevice close to the hillside of the limestone hill. The hill is isolated by maize fields. We, therefore, assess *Primulina flexusa* as Critically Endangered (CR C1), according to IUCN RedList Categories and Criteria (IUCN 2019). The CR category assessment of this new species is based on the distributional range that extends ca. 5 km^2^ around the type locality at present, as observed in the past two years.

#### Additional specimens examined.

*Primulina curvituba* B. Pan, L.H. Yang & M. Kang, **China**: Guangdong Province, Guangzhou City, cultivated in South China Botanical Garden, introduced from Guangxi Zhuangzu Autonomous Region, 25°11'31.83"N, 108°14'52.41"E, growing on the moist rock surfaces of limestone hills, 29 Jul 2016 (flowering), *Li-Hua Yang*, *YLH368* (holotype: IBSC!). *Primulina tenuituba* (W.T. Wang) Y.Z. Wang, **China**: Hunan Province, Yongshun County, Qingtianping, growing on the limestone cliff, 14 April 2013, *Hong-Wen Huang 40826* (CSFI!);Hunan Province, Longshan County, no detailed information, 11 April 2013, *Dai-Ke Tian*, *Yan Xiao*, *Yue Chen LS-1310* (CSH!); Hunan Province, Suining County, Huangsang National Natural Reserve, Huangsang Village, Dawanpeng, 413 m a.s.l., 20 April 2014, *Jian-Jun Zhou & Zong-Ping Song 1404145* (CSFI!); Hunan Province, Suining County, Huangsang National Natural Reserve, Pingxi Village, Banchong, 894 m a.s.l., 3 May 2014, *Jian-Jun Zhou & Zong-Ping Song 1405039* (CSFI!); Hunan Province, Suining County, Huangsang National Natural Reserve, Chiban Village, Yuanyang, 461 m a.s.l., 7 April 2013, *Jian-Jun Zhou & Dian Zhou 13024* (CSFI!); Hunan Province, Suining County, Huangsang National Natural Reserve, Dawantang Village, Da, 410 m a.s.l., 11 April 2013, *Jian-Jun Zhou & Dian Zhou 13136* (CSFI!); Hunan Province, Suining County, Huangsang National Natural Reserve, Chiban Village, Yuanyang, 487 m a.s.l., 11 April 2013, *Jian-Jun Zhou & Dian Zhou 13121* (CSFI!); Guizhou Province, Tongren City, Yangtou District, Jiulongdong, growing on the surface of rocks, 8 July 1988, *Wulingshankaochadui 1577* (PE!); Guizhou Province, Guiyang City, Dongshan, growing in the crevices of limestone hill, 8 May 1991, *De-Yuan Chen & Cheng-Gang Hu*, *s.n.* (PE!); same locality, 21 May 1989, *De-Yuan Chen*, *s.n.* (PE!); Sichuan Province, Daxian County, Pingchang, Heishui, 600 m a.s.l., 1 June 1978, *Pingchangdui 212* (SM!); Sichuan Province, Bazhong City, Daluo, Shedan, 700 m a.s.l., 8 March 1979, *Bazhongpuchadui 879* (SM!); Sichuan Province, Dazhu County, Zhuqi, Sanqi, Zhongfeng, 9 September 1978, *Dazhuxian 0803* (SM!); Sichuan Province, Nanjiang County, Yangba, 28 August 1978, 949 (SM!); Sichuan Province, Xiushan County (Chongqing City now), Shitang, 370 m a.s.l., 17 May 1979, 0349 (SM!).

**Figure 2.**
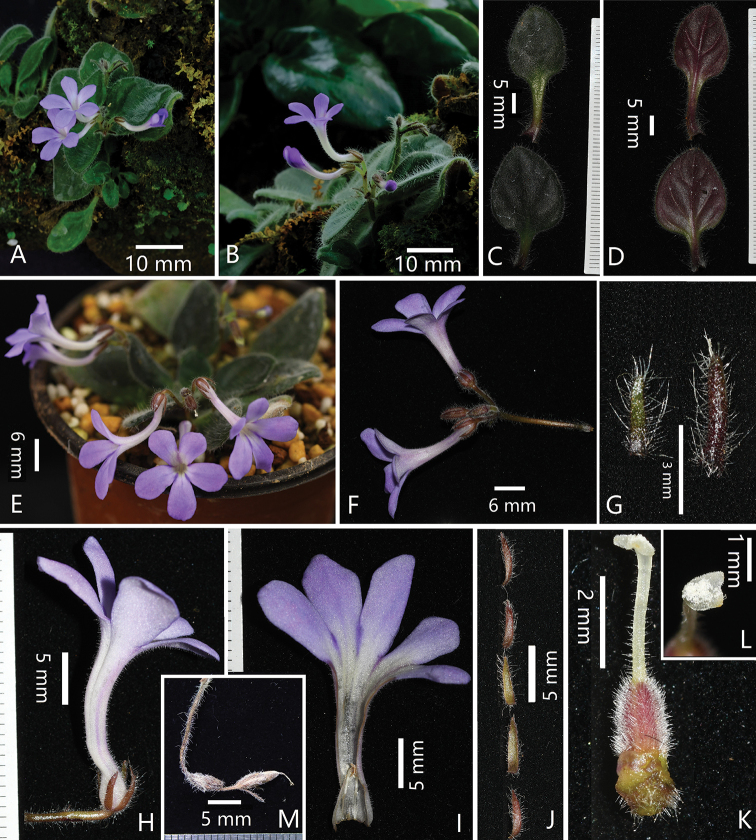
*Primulina flexusa* sp. nov. **A** habitat **B** habitat and flowering plant for showing curved corolla tube **C** adaxial surfaces of leaves **D** abaxial surfaces of leaves **E** cultivated potted plant in flowering **F** cyme **G** adaxial surfaces of bract (right) and bracteole **H** lateral view of corolla for showing curved corolla tube **I** opened corolla **J** calyx lobes **K** pistil **L** stigma **M** mature capsule.

#### Notes.

**Figure 3.**
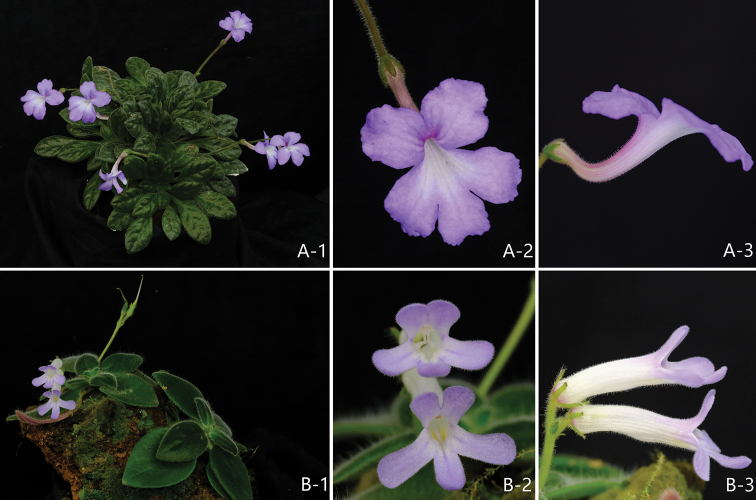
**A***Primulina curvituba***B***P. tenuituba*: **1** cultivated plants in flowering **2** frontal view of corolla **3** lateral view of corolla for showing the curved and straight corolla tube.

This new species is closely related to *Primulina curvituba* (Figure 3A), from which its vegetative and reproductive organs are obviously different, as stated in the diagnosis. The new species also resembles *P. tenuituba*; however, the latter has no curved corolla tube (Figure 3B). The detailed morphological differences among the three species are summarized in Table 1.

**Table 1.** Detailed comparisons among *Primulina flexusa*, *P. curvituba*, and *P. tenuituba*.

Characters	*P. flexusa*	*P. curvituba*	*P. tenuituba*
Leaf blade			
Shape	ovate to broadly ovate	elliptical to linear-elliptical	ovate to suborbicular
Size	1.6–2.0 × 1.3–1.8 cm	1.4–3.3 × 0.9–1.5 cm	1–3.2 × 0.8–2.5 cm
Indumentum	pubescent and pilose on abaxial surface, puberulent on the adaxial surface	with both surfaces densely white pubescent	appressed pubescent to appressed pilose
Margin	entire and ciliate	entire and revolute	entire to repand-crenate
Cyme			
Number / per plant	2–4	6–11	2–4
Peduncle length	1.5–2.4 cm	3–6.5 cm	0.6–1.4 cm
Bracts			
Shape	oblong	lanceolate	narrowly triangular to lanceolate
Size	ca. 3.8 × 0.7 mm	2.0–3.5 × 1.0–1.5 mm	0.8–3 × 0.3–1 mm
Indumentum	adaxially nearly glabrous, abaxially densely pilose	adaxially glabrescent, abaxially densely white pubescent	adaxially glabrescent, abaxially puberulent to pilose
Pedicel			
Length	6.5–8 mm	20–30 mm	2–5.5 mm
Indumentum	puberulent	densely pubescent	densely spreading puberulent to pilose
Calyx lobes size	4–5 × 0.7–0.9 mm	2–3 × 1.0–1.5 mm	4.5–5.5 × 0.8–1.2 mm
Corolla indumentum	outside covered extremely short glandular-puberulent hairs, inside nearly glabrous	outside pubescent, inside glabrescent	outside sparsely puberulent, inside puberulent below the abaxial lip
Corolla tube	infundibuliform, slightly curved downwards at the base (ca. 4 mm from the base), then gradually bent forwards	infundibuliform, laterally compressed at the mouth, strongly curved downwards at the base (5 – 7 mm from the base), then bent forwards	cylindric, straight
Filament			
Length	ca. 3 mm	5–6 mm	4.5–5.5 mm
Indumentum	glabrous	glandular-pubescent	Glabrous
Staminodes number and length	3, lateral ones ca. 3.5 mm, the middle one ca. 0.7 mm	3, lateral ones 1–2 mm, middle one ca. 1 mm	2, 0.5–0.8 mm
Pistil			
Length	ca. 5 mm	7–8 mm	19–22 mm
Ovary			
Length	ca. 1.4 mm	5–6 mm	3.2–6 mm
Shape	ovoid	cylindrical	Cylindrical
Stigma	slightly 2-parted at apex	undivided at apex	2-parted at apex
Capsule			
Length	ca. 6.5 mm	10–15 mm	2–2.8 mm
Shape	ovoid	linear	

## Supplementary Material

XML Treatment for Primulina flexusa
